# Epidemiology of Coronavirus Disease in Gansu Province, China, 2020

**DOI:** 10.3201/eid2606.200251

**Published:** 2020-06

**Authors:** Jingchun Fan, Xiaodong Liu, Weimin Pan, Mark W. Douglas, Shisan Bao

**Affiliations:** School of Public Health, Gansu University of Chinese Medicine, Lanzhou, China (J. Fan);; Institute of Immunization and Prevention Management, Shandong Center for Disease Control and Prevention, Jinan, China (X. Liu);; Gansu Provincial Center for Disease Control and Prevention, Lanzhou (W. Pan);; Centre for Infectious Diseases and Microbiology, Westmead Hospital, Sydney, New South Wales, Australia (M.W. Douglas);; Storr Liver Centre and Marie Bashir Institute for Infectious Diseases and Biosecurity, University of Sydney, Sydney (M.W. Douglas);; School of Medical Sciences and Bosch Institute, Charles Perkins Centre, University of Sydney, Sydney (S. Bao)

**Keywords:** SARS-CoV-2, severe acute respiratory syndrome coronavirus 2, COVID-19, outbreaks, Wuhan, Gansu Province, China, viruses, coronavirus, 2019 novel coronavirus disease, respiratory infections, zoonoses

## Abstract

To determine the epidemiology of coronavirus disease (COVID-19) in a remote region of China, far from Wuhan, we analyzed the epidemiology of COVID-19 in Gansu Province. From January 23 through February 3, 2020, a total of 35 (64.8%) of 54 reported cases were imported from COVID-19–epidemic areas. Characteristics that differed significantly during the first and second waves of illness in Gansu Province were mean patient age, occupation, having visited epidemic areas, and mode of transportation. Time from infection to illness onset for family clusters was shorter in Gansu Province than in Wuhan, consistent with shortened durations from onset to first medical visit or hospitalization. Spatial distribution pattern analysis indicated hot spots and spatial outliers in Gansu Province. As a result of adequate interventions, transmission of the COVID-19 virus in Gansu Province is decreasing.

The outbreak of coronavirus disease (COVID-19) was first reported on December 31, 2019, in Wuhan, China (*1*). Within a few weeks, the virus had spread rapidly throughout China and within 1 month to several other countries, including Italy (*2*), the United States (*3*), and Germany (*4*). Difficulty controlling such aggressive spread resulted partly from the size of Wuhan, which has a full-time population of >9 million and a transient population of an additional 5.10 million, for a total population of ≈14 million (*5*,*6*). Wuhan is located in central China and has a wide range of transportation links, including airplanes, trains, interstate buses, and private transportation. In an attempt to reduce virus transmission, on January 23, 2020, authorities locked down Wuhan, but by that time, ≈5 million persons had already left (*7*). Reasons for leaving included returning to hometowns for the Chinese New Year (most persons) or leaving for holidays, but some left because of fear of COVID-19 (*7*). Consequently, COVID-19 has now been identified in every province or autonomous region in China, although the highest number of cases is still in Wuhan (*7*).

Gansu Province is located in northwestern China, and as of February 3, 2020, the number of COVID-19 cases identified has been small, most in persons coming from Wuhan. As of January 23, the date of the Wuhan lockdown, the Gansu Provincial Centre for Disease Control and Prevention (Lanzhou, China) had identified only 2 cases of COVID-19 in Lanzhou, the capital of Ganzu Province: 1 patient had traveled from Wuhan and another had been in contact with persons from Wuhan (*8*). Within 2 weeks, however, 54 cases of COVID-19 in Gansu Province were confirmed, indicating the seriousness of this outbreak and triggering the alarm for the Gansu Province government to make it mandatory that face masks be worn in public places from January 28 until further notice (*9*). From a public health point of view, the epidemiology of the COVID-19 event in Lanzhou (*10*), a relatively remote region of China, may provide critical information to help control the spread of this disease to other provinces and countries. We therefore explored the epidemiology of COVID-19 in Gansu Province, remote from the outbreak epicenter in Wuhan.

## Materials and Methods

We split the 12-day study period in half. The early period began January 23, the date of the first confirmed case of COVID-19 reported in Gansu Province, and continued through January 28, the date when the Gansu government decreed it mandatory to wear face masks in public places. The late period, the subsequent 6 days, extended from January 29 through February 3. To analyze the epidemiology of the COVID-19 outbreak in Gansu Province, we compared cases diagnosed during the early and late periods. Our aim was to compare groups to determine if the restriction order was effective for controlling transmission. The definition of primary versus secondary cases refers to whether persons traveled from Wuhan (primary) or never left Gansu Province (secondary). The aim of this distinction was to explore potential transmission.

The first wave of infection was seen in persons who arrived from Wuhan, whereas later infections resulted from virus transmission from the first group of persons. To compare the demographic and clinical characteristics of persons with primary and secondary infection, we collected data about transmission and family clusters, including the date of return from epidemic areas, first day of close contact, date of symptom onset, date of first medical visit and hospitalization, and relationships between patients with primary and secondary cases.

### Setting

**Figure 1:**
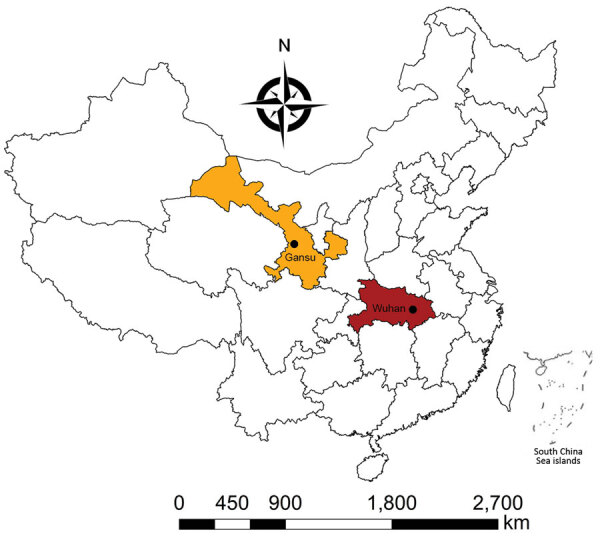
Location of Gansu Province and Wuhan, China. Circles indicate capital cities.

**Figure 2:**
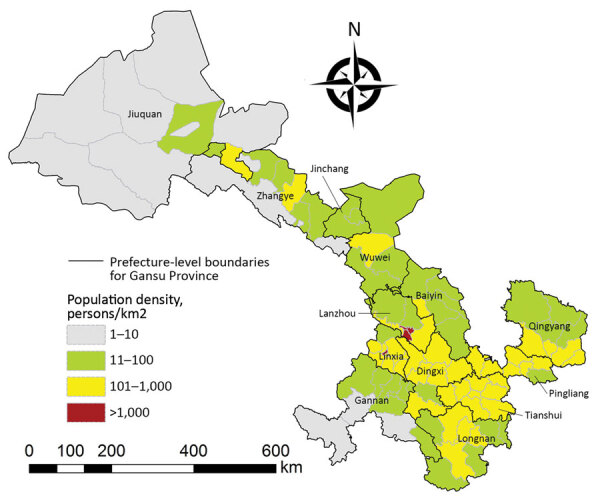
Population density of Gansu Province, China, in 2018.

Gansu Province (32°31′N–42°57′N, 92°13′E–108°46′E ﻿) is located in northwestern China (Figure 1). Gansu is the seventh largest province in China, comprising 12 prefecture-level cities and 2 autonomous prefectures (86 counties and districts), with a total land area of 454,000 km^2^ and a population of 26,257,100 in 2019 (*11*). It is a long, handle-shaped province, and Lanzhou is located on the Yellow River. The complex landforms of Gansu Province include mountainous regions, plateaus, plains, river valleys, and desert. With a population of 3.8 million and 13,100 km^2^, the population density of Lanzhou is 287 persons/km^2^ (Figure 2), although Lanzhou is classified as a third-tier city in China (*10*).

### Materials

COVID-19 diagnoses in Gansu Province from January 23 through February 3, 2020, were confirmed in the laboratory of Gansu Provincial Centre for Disease Control and Prevention (*12*). Suspected cases of COVID-19 infection were identified in hospitals and confirmed in the same laboratory by specific nucleic acids. We collected demographic data, including patient sex, age, occupation, place of residence, and exposure history, from the official website of the Gansu Provincial Center for Disease Control and Prevention (http://gscdc.net). 

Within each prefecture or prefecture-level city in Gansu Province are districts, counties or autonomous counties, and county-level cities. For this study, we classified all counties and county-level cities as counties for simplicity and for data analysis. To conduct a geographic information system (GIS)–based analysis on the spatial distribution of COVID-19 cases, we applied the county-level polygon map (county layers) at 1:250,000 scale from Data Sharing Infrastructure of Earth System Science (http://www.geodata.cn), on which we generated a county-level layer containing information regarding latitudes and longitudes of cases in county layers (central points) of each county.

### Statistical Analyses

The 54 patients were assigned numbers from 1 to 54 according to time of diagnosis. The statistical descriptions included demographic characteristics, exposure history, whether the cases were primary or secondary, and potential spread of disease. Because the ages of case-patients were not normally distributed, we performed nonparametric Brown-Mood tests to compare medians between early and late cases. For expected cell sizes <5, we used the Fisher exact test to compare the frequency between or among groups; otherwise, we used the χ^2^ test. We estimated days from illness onset to first medical visit and days from illness onset to hospitalization by fitting a Weibull distribution to the dates of illness onset, first medical visit, and hospital admission (*13*).

### GIS Mapping and Spatial Analyses

We geocoded all COVID-19 cases and matched them to the county-level layers of polygon and point by administrative codes by using ArcGIS 10.2.2 software (https://www.arcgis.com). To explore the spatial distribution pattern of COVID-19 cases on the county level during the study periods, we applied local indicators of spatial association (LISA [*14*]). Using LISA, we could identify the type and degree of spatial clustering, including significant hot spots (high-high), cold spots (low-low), and spatial outliers (high-low and low-high) between a given location and surrounding spatial units by calculating the local Moran’s I (*14*,*15*). We used the Z statistic to determine the significance of clustering based on a significance level of 0.05. A significant positive Z indicates high-value regions surrounded by high-value regions (high-high) or low value regions surrounded by low-value regions (low-low). A significant negative Z indicates high-value regions surrounded by low-value regions (high-low) or low-value regions surrounded by high-value regions (low-high) (*16*).

### Ethics Approval

Our study was approved by the institutional review board, Gansu University of Chinese Medicine. We collected data from the official website of Gansu Provincial Center for Disease Control and Prevention, which was considered exempt from approval.

## Results

### Patient Characteristics

**Figure 3:**
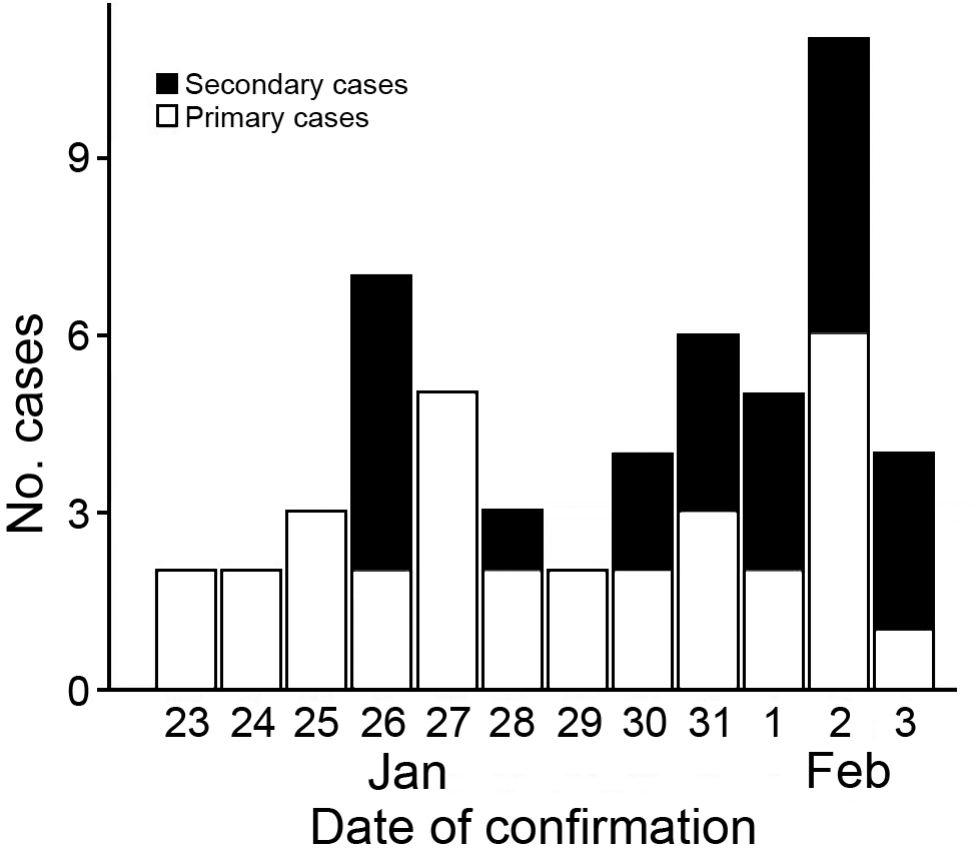
Time series of coronavirus disease case identification in Gansu Province, China, 2020.

Of the total 54 cases of COVID-19 in Gansu Province, 35 were imported primary and 19 were indigenous secondary cases. Serious/critical illness was experienced by 6 (17.1%) of the 35 patients with primary cases and by 3 (15.7%) of the 19 patients with secondary cases; the difference was not significant (p = 0.899). A total of 24 cases were reported during the early period and 30 during the late period (Figure 3). On January 26, the first secondary case of COVID-19 was identified in Longnan, a city located in southern of Gansu Province. The youngest patient was 1 year and 8 months of age; the oldest was 94 years of age. We used Cox proportional hazards regression to correct for covariates but found no significant differences between the early and late groups. No survival pattern could be calculated because, to date, no COVID-19–associated deaths in Gansu Province have been reported. 

Comparing patients who sought care in the early and late periods, we found significant differences for age (median age 34 years for the early period vs. 48 years for the late period; p = 0.014) and for occupation (more laborers in the early period vs. more retired persons in the late period; p = 0.009) but not for patient sex or whether the patients lived in urban or rural areas (Table 1). In addition, more case-patients in the early period had visited epidemic areas (p = 0.043) and returned to their destination (p = 0.024) than had patients in the late period.

**Table 1:** Characteristics of patients with coronavirus disease, Gansu Province, China, 2020*

Characteristics	Jan 23–28, n = 24	Jan 29–Feb 3, n = 30	p value
Median age (range), y	34 (20–83)	48 (1.67–94)	0.014
Age group, no./total no. (%), y			0.821
<18	0/24	1/30 (3)	
18–64	22/24 (92)	25/30(83)	
>65	2/24 (8)	4/30(13)	
Sex			0.854
M	11/24 (46)	13/30 (43)	
F	13/24 (54)	17/30 (57)	
Occupation			0.009
Cadres and professionals	3/24 (8)	8/30 (27)	
Laborers	7/24 (29)	3/30 (10)	
Farmers	3/24 (13)	0/30	
Business service providers	3/24 (13)	2/30 (7)	
Students	2/24 (8)	3/30 (10)	
Scatter children† and retires	4/24 (17)	16/30 (47)	
Healthcare workers	2/24 (8)	0/30	
Urban residence	14/24 (58)	20/30 (67)	0.529
Primary case	19/24 (79)	17/30 (56)	0.081
Exposure to epidemic area			0.043
Hubei Province	16/24 (66)	14/30 (47)	
Other area	3/24 (13)	2/30 (7)	
No exposure to Hubei/other area	5/24 (21)	14/30 (46)	
Main mode of transportation			0.193
Airplane	6/24 (25)	3/30 (10)	
Train	7/24 (29)	8/30 (27)	
Airplane and train	6/24 (25)	5/30 (17)	
Local, did not leave Gansu Province	5/24 (21)	14/30 (46)	
Frequency of transfer between transportation modes			0.024
<3	7/24 (29)	11/30 (37)	
>3	12/24 (50)	5/30 (16)	
Local, did not leave Gansu Province	5/24 (21)	14/30 (47)	

*Data are no./total no. (%) unless otherwise indicated. †Children who stayed at home, did not attend childcare facilities.

### Key Time-to-Event Distributions of COVID-19 

**Figure 4:**
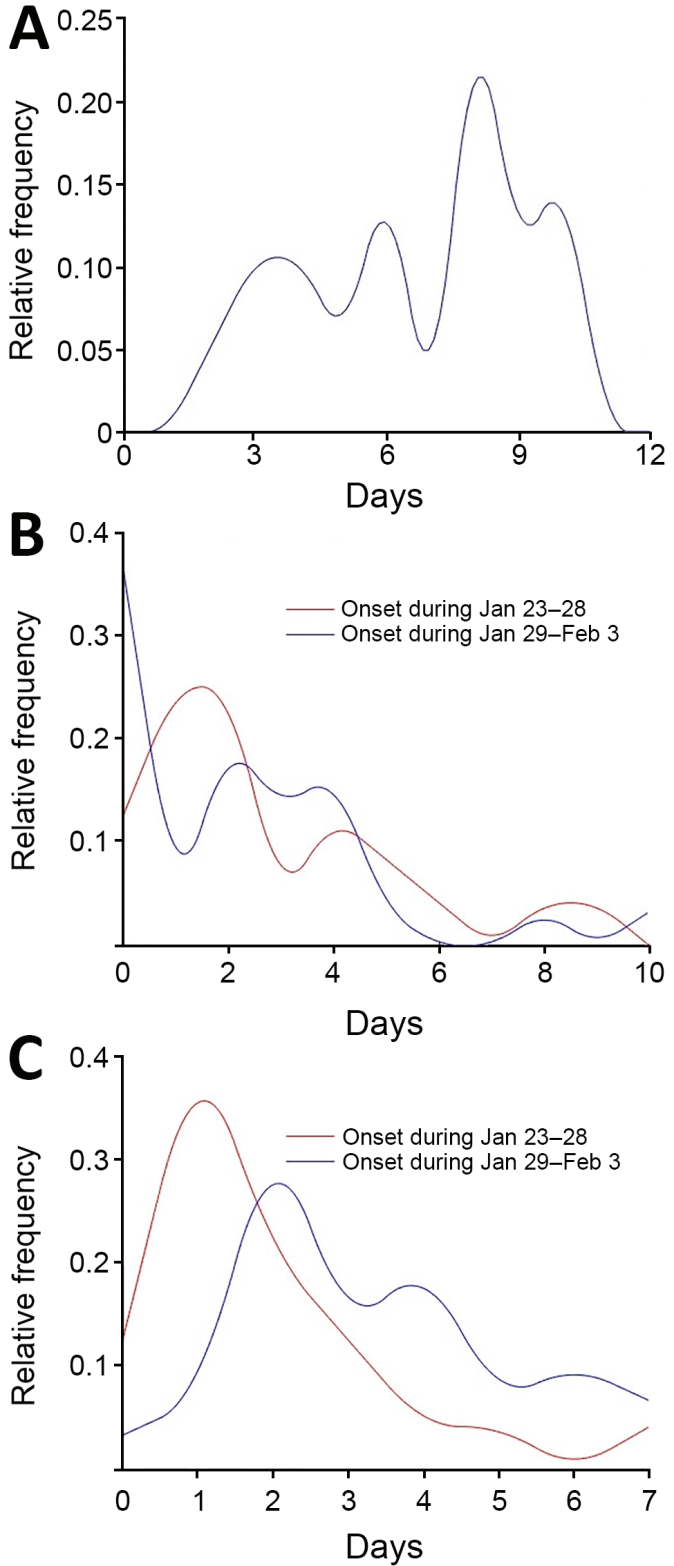
Key time-to-event distributions of coronavirus disease cases in Gansu Province, China, 2020. A) Incubation period (i.e., days from infection to illness onset). B) Days from illness to first medical visit. C) Days from illness to hospitalization.

For the 54 cases of COVID-19 diagnosed in Gansu Province from January 23 through February 3, the days from illness onset to first medical visit ranged from 0 to 10 and the days from first medical visit to hospitalization ranged from 0 to 7. The days from infection to symptom onset for 19 case-patients in a family cluster, confirmed by epidemiologic survey, ranged from 2 to 10 days (mean 6.7 days); the 95th percentile of the distribution was 12.5 days (95% CI 9.2–18 days) (Table 2; Figure 4, panel A). Among the 24 COVID-19 cases confirmed in the early period, the estimated mean time from illness onset to first medical visit was 2.8 days (95% CI 1.7–3.8); that is, there was a trend toward longer delay for case-patients in the early period than the mean of 2.3 days (95% CI 1.3–3.2) for the 30 case-patients in the late period (Figure 4, panel B), but this difference did not reach statistical significance (p>0.05). The mean duration from first medical visit to hospital admission was 1.9 days (95% CI 1.2–2.6 days) among 24 case-patients with illness onset during the early period, shorter than among 30 case-patients with illness onset during the second period, among whom the mean duration from first medical visit to hospital admission was 3.3 days (95% CI 2.7–4.0 days) (Figure 4, panel C).

**Table 2:** Interval between primary and secondary cases in 6 family clusters of coronavirus disease, Gansu Province, China, 2020*

Family cluster patient no.	No. primary cases	No. secondary cases	Date of close contact	Date of symptom onset	Serial interval, d†
1	1	9	Jan 18	22 Jan	4
2	1	26	Jan 18	26 Jan	8
3	1	27	Jan 18	26 Jan	8
4	4	14	Jan 19	25 Jan	6
5	4	46	19 Jan	27 Jan	8
6	6	10	15 Jan	21 Jan	6
7	6	11	15 Jan	21 Jan	6
8	6	12	15 Jan	24 Jan	9
9	6	13	15 Jan	24 Jan	9
10	6	23	15 Jan	23 Jan	8
11	29	39	25 Jan	30 Jan	5
12	29	47	22 Jan	26 Jan	4
13	Family gathering‡	43	26 Jan	28 Jan	2
14	Interstate business‡	44	15 Jan	23 Jan	8
15	36	48	20 Jan	23 Jan	3
16	36	49	21 Jan	24 Jan	3
17	36	50	20 Jan	30 Jan	10
18	36	51	20 Jan	30 Jan	10
19	36	52	20 Jan	30 Jan	10

*Because incidence was derived from a family gathering, dates of close contact and symptom onset are similar. †Days between close contact and symptom onset. ‡Local residents for whom primary cases could not be traced.

### Spatial Distribution of COVID-19 

**Figure 5:**
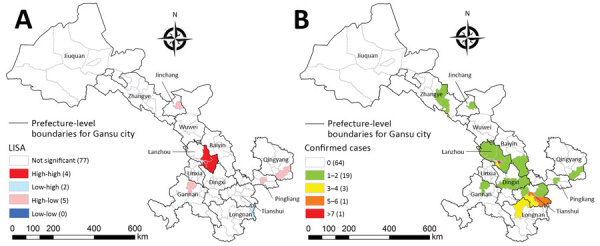
Distribution of reported coronavirus disease cases (A) and local indicators of spatial association cluster map (B) for Gansu Province, January 23–February 3, 2020. Numbers in parentheses indicate number of counties.

The 54 COVID-19 cases reported in Gansu Province from January 23 through February 3 were distributed in 11 prefectures and 24 counties. The 2 cities with the most COVID-19 case-patients were Lanzhou (26 cases) and Tianshui (8 cases). The area with the most COVID-19 cases was Chengguan District, the political and economic center of Lanzhou (Figure 5, panel A). Patients with secondary cases seem to have been infected by persons with primary cases; we identified 6 family clusters: 3 in Lanzhou, 1 in Tianshui, 1 in Longnan, and 1 in Linxia. LISA analysis demonstrated hot spots (high-high) and outliers (high-low and low-high). The high-high cluster included 4 counties, accounting for 4.55% of all counties, mainly distributed in the eastern capital city of Lanzhou. High-low and low-high outliers were sporadically distributed in eastern-central Gansu Province (Figure 5, panel B).

## Discussion

Our data suggest that most COVID-19 cases that occurred in the first 6 days (January 23–28, 2020) were imported from Wuhan (the coronavirus epicenter). Most secondary cases occurred in the second 6 days (January 29–February 3, 2020).

In Gansu Province, the patients identified in the early period were younger than those identified in the late period, but overall the 54 patients were younger (median age 38 years) than patients identified in the early stage of the outbreak in Wuhan (median age 59 years) (*13*). This finding is most likely because Wuhan is where the outbreak originated in China (*17*). Patients from the early period were younger and had mainly imported primary cases, possibly because most of them were labor workers in Wuhan who traveled back to Gansu Province; in contrast, a greater proportion of patients in the late period were senior retirees with secondary indigenous cases. The proportions of COVID-19 patients who had visited an epidemic area and the frequencies of transfer were 3 times greater during the early than the late period, suggesting that exposure risk would decrease from epidemic areas and public places as the epidemic spread. The current situation may result from the travel bans from Wuhan or other places as well as most persons’ awareness and education about COVID-19 (*18*), but human transportation had spread the virus before Wuhan was locked down. (*19*).

In our study, distribution of illness by sex did not differ significantly between the early and late periods, but female patients predominated slightly. In contrast, Chen and colleagues reported more male than female patients in the Wuhan outbreak (*20*). One possible explanation is that in Wuhan, men dominate the labor market and probably had close contact with the seafood market in Wuhan (the source of the outbreak), resulting in more male patients in the Wuhan study. In contrast, the COVID-19 outbreak in Gansu was 2 weeks later, during the Chinese New Year festival, in which family members of both sexes participated. Thus, our data further support the need for broad infection control measures in surrounding areas, irrespective of age, sex, place of residence, or mode of transportation.

The first case of secondary infection was identified in a patient who had had close contact with the first case-patient with primary COVID-19 in Gansu Province, after a lag of 4 days. Subsequently, 6 family clusters were identified over 12 days. These data confirm direct human-to-human transmission of the COVID-19 virus, severe acute respiratory syndrome coronavirus 2 (SARS-CoV-2), through close contact (*21*). This finding supports the government restriction of persons gathering in large groups, to reduce or minimize virus transmission. The 95% percentile range for the incubation period found in our study is consistent with the findings for Wuhan, which almost completely overlap (*13*), suggesting that within this short time, SARS-CoV-2 has not mutated sufficiently to affect incubation time (*22*). 

The proportion of persons who visited a medical center or hospital within 2 days from illness onset was 61% in Gansu Province, compared with 27% in Wuhan (*13*). In addition, 68% of patients in Gansu Province were hospitalized within 5 days after illness onset, whereas only 11% of patients in Wuhan were hospitalized in the early stages of the disease (*13*). These differences between Gansu Province and Wuhan may partially result from differences in numbers of staff and medical resources between Wuhan and Gansu Province, relative to the number of COVID-19 patients. Another potential factor is advanced warning about COVID-19 in Gansu Province via various channels. Although knowledge about COVID-19 was minimal at the beginning of the outbreak, the seriousness of COVID-19 was well broadcast throughout China, including Gansu Province, after the initial outbreak. Compared with the initial stage of the COVID-19 outbreak in Wuhan, the reduced incidence of COVID-19 in Gansu Province over this period may be associated with reinforcement of mandatory control and prevention interventions, patients voluntarily seeking medical assistance after being educated by the media, or possibly both.

Our study demonstrates a significant spatial heterogeneity of COVID-19 cases in Gansu Province over this 2-week period; cases were mostly concentrated in Lanzhou and surrounding areas. LISA analysis findings are in agreement with the spatial distribution of COVID-19 at the county levels of Gansu Province. This analysis confirms that the distribution of cases was not random: hot spots were mainly restricted to the Chengguan District of Lanzhou, the most densely populated and most developed area (*11*). This case aggregation is closely associated with the development characteristics of Gansu Province, which is at the high end of economic, medical, population, and cultural development. Consequently, Chengguan District was the most common pathway for most persons returning from Wuhan and other cities, who end up in or transit through Lanzhou. Land formations in Gansu Province limit travel, particularly in mountainous rural areas, which may have slowed down or restricted the spread of COVID-19 across Gansu Province. For example, cases have been reported in Wuwei, a city in a remote, mountainous, hard-to-reach area in Gansu Province. Our findings are supported by other studies, which demonstrate a close correlation with population and economy in other major cities of China where outbreaks of COVID-19 occurred within 1–2 weeks of the original outbreak in Wuhan (*23**–**25*).

Although case numbers are small, in Gansu Province the severity of COVID-19 illness did not differ between persons with primary and secondary cases, suggesting that the virus does not mutate to decrease virulence during ongoing transmission. This finding is consistent with recent reports from Italy and the United States, which suggest that mutations that reduce transmission do not spontaneously develop in SARS-CoV-2 (*2*,*3**)*. In Gansu Province, we observed fewer severe clinical cases of COVID-19, lower rates of patients in critical condition (13%), and no deaths, compared with Wuhan, where ≈30% patients were admitted to intensive care and 4% died (*26*). One possible explanation is insufficient medical staff and resources in Wuhan to deal with the large outbreak and only extremely serious or critical patients being hospitalized, in contrast to Gansu Province, where relatively more medical staff and resources were available. Furthermore, a large proportion of the Wuhan population who left the city before the lockdown were migrant workers, commercial personnel, and college students; the population remaining in Wuhan after the lockdown therefore comprised mostly elderly persons with compromised immunity (*27*). The incubation period in Gansu Province was longer than that in Wuhan (3–6 days for Wuhan) (*28*,*29*). This finding may be the result of the government’s intensive provision of information for the whole nation about controlling and preventing COVID-19.

A limitation of our study is the difficulty of calculating county-level incidence or estimating the risk factors affecting SARS-CoV-2 transmission in Gansu Province because of the relatively small number of cases and the short study period (i.e., 12 days), which may not reflect the entire epidemiology of the virus in Gansu Province and will only become clear over time. Whether the outbreak will be controlled soon, and if so when, will be answered in due course.

In conclusion, our study demonstrates the epidemiology of a relatively small-scale outbreak of COVID-19 outside of Wuhan. Our cohort included primary cases from Wuhan and subsequent secondary cases, including several family clusters. Such information should be useful for other regions and countries to help combat the spread of this lethal disease (*30*) by informing the development of more effective local infection control policies and recommendations.
